# Effects of exercise and metformin on the prevention of glucose intolerance: a comparative study

**DOI:** 10.1590/1414-431X20153904

**Published:** 2015-09-29

**Authors:** C. Molena-Fernandes, C. A. Bersani-Amado, Z. M. Ferraro, L. J. Hintze, N. Nardo, R. K. N. Cuman

**Affiliations:** 1 Colegiado de Educação Física, Universidade Estadual do Paraná, Paranavaí, PR, Brasil; 2 Departamento de Farmácia e Farmacologia, Universidade Estadual de Maringá, Maringá, PR, Brasil; 3 Children’s Hospital of Eastern Ontario Research Institute, Healthy Active Living and Obesity Research Group, Ottawa, ON, Canada; 4 Division of Maternal-Fetal Medicine, Obstetrics and Gynecology, The Ottawa Hospital, Ottawa, ON, Canada; 5 Departamento de Educação Física, Universidade Estadual de Maringá, Maringá, PR, Brasil

**Keywords:** Exercise, Glucose intolerance, Dexamethasone, Wistar rats, Diabetes mellitus, Prevention and control

## Abstract

We aimed to evaluate the effects of aerobic exercise training (4 days) and metformin exposure on acute glucose intolerance after dexamethasone treatment in rats. Forty-two adult male Wistar rats (8 weeks old) were divided randomly into four groups: sedentary control (SCT), sedentary dexamethasone-treated (SDX), training dexamethasone-treated (DPE), and dexamethasone and metformin treated group (DMT). Glucose tolerance tests and *in situ* liver perfusion were undertaken on fasting rats to obtain glucose profiles. The DPE group displayed a significant decrease in glucose values compared with the SDX group. Average glucose levels in the DPE group did not differ from those of the DMT group, so we suggest that exercise training corrects dexamethasone-induced glucose intolerance and improves glucose profiles in a similar manner to that observed with metformin. These data suggest that exercise may prevent the development of glucose intolerance induced by dexamethasone in rats to a similar magnitude to that observed after metformin treatment.

## Introduction

Diabetes mellitus (DM) is characterized by a hyperglycemic postprandial state due to a partial or total absence of insulin released by pancreatic beta cells, peripheral resistance to insulin, or both (1). Type 2 diabetes (DM2) comprises the highest incidence among the different classes of DM, and accounts for approximately 90% of cases (2,3). The number of DM2 cases has increased rapidly and has reached epidemic proportions worldwide (4,5). Hence, DM has become a major public health problem. The prevalence of DM2 in the general population, whether in developed or developing countries, varies between 3% and 7%. About 177 million people have DM worldwide, and this number is expected to double by 2030 (3,5).

The onset of DM2 occurs over a variable time period and progresses from an intermediate stage known as “impaired glucose tolerance” or “glucose intolerance” (1,6) and may evolve to clinical presentation of DM. Glucose intolerance, a major risk factor for development of DM2, and rate of DM progression in people with glucose intolerance, varies from 4% to 8% a year in different populations (7). There are several known risk factors for development of DM2: advanced age, obesity, unhealthy dietary habits, and lack of physical activity (8). Indeed, lifestyle modification is cited as the “gold standard” management for inhibiting development of DM2 in high-risk individuals with glucose intolerance. Clinical trials have reported the beneficial effects of lifestyle intervention programs in high-risk diabetes-prone individuals (9-11). Data from the Diabetes Prevention Program Group (10) and American Diabetes Association (1) demonstrated that changes in eating habits and increased physical activity resulted in a diminished risk of DM2 progression (60%) in individuals with glucose intolerance after 3 years of intervention. Similarly, other studies have demonstrated that physical activity, decreased obesity, and insulin resistance can prevent DM2 (10-12). However, the effect of physical activity compared with metformin on the improvement of glucose tolerance has not been investigated thoroughly (5,13,14).

Experimental data have demonstrated that aerobic physical exercise reduces levels of glucose in the tissues of control and diabetic rats (15-20). However, few studies assessing the effects of physical exercise in experimental models of acute hyperglycemia have been conducted. Thus, the influence of aerobic physical exercise and metformin on development of acute glucose intolerance in rats was evaluated.

## Material and Methods

### Animals

Adult male Wistar rats (200-250 g) were housed at 22±2°C under a 12-h dark-light cycle, and given a standard pelleted diet and water *ad libitum*throughout the experimental period. The experimental protocol was approved by the Animal Ethics Committee of Universidade de Maringá (CEEA, #033/2007). The animals included in the study were weighed every day during the experimental period.

### Experimental procedures

**Figure 1:**
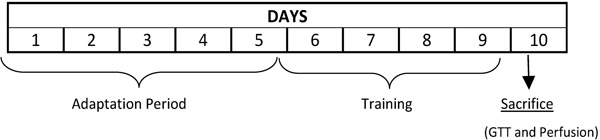
Experimental design. GTT: glucose tolerance test.

Animals (n=42) were divided randomly into four experimental groups. The sedentary control group (SCT; n=8) was treated with 0.9% NaCl solution (*sc*, for 4 days) without any swimming activity. The sedentary dexamethasone group (SDX; n=12) was treated with 0.1 mg/kg dexamethasone (DEXA, *sc,* daily, for 4 days) without any swimming activity. The DEXA-treated and physical exercise group (DPE; n=10) was treated with 0.1 mg/kg DEXA (*sc,* daily, for 4 days) with swimming activity (4 days, 1 h/day). Finally, the DEXA and metformin (MET) treated group (DMT; n=12) was treated with 0.1 mg/kg DEXA (*sc,*daily, for 4 days) and MET (300 mg·kg^−1^·day^−1^; gavage, for 4 days). All treatments ended 1 day before the experiments (Figure 1).

### Model of dexamethasone-induced acute hyperglycemia

To mimic the disease observed in prediabetes and DM2, DEXA was administered to animals by subcutaneous injection (0.1 mg/kg body weight) during 4 days to induce acute hyperglycemia. Control animals were given 0.9% NaCl during 4 days. A fasted glucose tolerance test (GTT) was undertaken on animals to determine glucose intolerance.

### Intravenous GTT

All rats were fasted for 24 h and then anesthetized (sodium pentobarbital, 40 mg/kg body weight, *ip*). After laparotomy, sequential blood samples were collected from the abdominal aorta immediately before glucose challenge (0.5 g glucose/kg body weight, *iv*) as well as 5, 15, 30, and 60 min thereafter. After centrifugation at 1350 *g* for 5 min at 4°C, 20 µL of serum was used immediately for glucose with a Gold Analisa Diagnóstica kit (Gold Analisa Diagnóstica, Brazil). The within-assay coefficient of variation was 1.2% and the between-assay coefficient of variation was 2.7%.

### *In situ* liver perfusion

Male albino Wistar rats (n=42; 180-220 g) were fed *ad libitum*with a standard laboratory diet (Purina¯, Brazil). Food was withdrawn 18 h before liver-perfusion experiments. Rats were manipulated according to international laws for ethical care and use (European Communities Council Directive of 24 November 1986, 86/609/EEC), conforming to national guidelines. Animals were anesthetized using sodium pentobarbital (50 mg/kg, *ip*) for the surgical procedure for liver isolation. Basal blood samples were collected before induction of any surgical procedure, and the non-recirculating method was executed, as described previously (21). In brief, after cannulation of the portal and cava veins, the liver was positioned in a plexiglass chamber and a peristaltic pump maintained constant flow. For the surgical procedure, the perfusion fluid was Krebs-Henseleit bicarbonate buffer (pH 7.4) containing 25 mg% bovine-serum albumin, saturated with a mixture of oxygen and carbon dioxide (95:5) by a temperature-regulated (37°C) membrane oxygenator before liver penetration by a cannula inserted in the portal vein. The perfusion flow was constant in each individual experiment. It was adjusted between 30 mL/min and 35 mL/min depending on liver weight. Samples of the effluent perfusion fluid were collected at 5-min intervals and analyzed for glucose concentration by the glucose-oxidase method. L-glutamine (5 mM) added to the perfusion fluid was used in all perfusion experiments as a substratum to gluconeogenesis parameters. Glucose concentration in serum or perfusate was determined by the glucose oxidase method (Gold Analisa Diagnóstica). Serum glucose is reported as mg/dL and in the perfusate as µmol·min^−1^·g^−1^.

### Physical exercise protocol (swimming)

The exercise protocol comprised daily 60-min swimming sessions during 4 days. A load equivalent to 5% of the body weight of the animal was attached to its tail. This protocol is considered to be a low-to-moderate intensity activity of long duration or, rather, a level of exercise sufficient to stimulate the onset of physiologic adaptations (22,23).

Individual swimming sessions were carried out in a 250 L rectangular water tank. Animals were separated inside the tank by polyvinyl-chloride pipes, and the water temperature was controlled by a thermostat maintained at 29±2°C. This temperature is thermally neutral and reduces temperature-induced stressors caused by temperatures above or below that of the environment. The water was changed after each exercise session to avoid contamination by feces and urine excreted during training.

Swimming was chosen as the exercise protocol because it is used frequently in rodent models for this field of study. It also provides lesser psychological stress to animals compared with that induced by a treadmill exercise (24).

Animals underwent a 5-day adaptation period: adaptation in liquid medium on the first day (15 min, without a load); 30 min of swimming without a load on the second day; 60 min without a load on the third day; 30 min with loads equivalent to 5% of body weight attached to the tail on the fourth day and, lastly, adaptation to 45 min and a load of 5% on the fifth day. Dexamethasone treatment commenced from the sixth day of training. Animals swam during a 4-day period, and the tests described above were then carried out on the fifth day (GTT and liver perfusion).

### Statistical analysis

Results are reported as means±SE. Between-group differences were analyzed using one-way analysis of variance (ANOVA) followed by Tukey's test for multiple comparisons to identify where potential differences existed. Paired *t*-tests were used to identify significant weight-related differences that emerged as a result of each treatment. P<0.05 was considered significant. Statistical analysis was carried out using GraphPad Prism¯ v5.0 (Microsoft, USA). The area under the curve (AUC) was calculated using the AUC feature in this software.

## Results

Although the control group did exhibit changes in body weight, a significant decrease in body weight was reported in the remaining groups at the end of the experiment. This decrease was greater for sedentary and dexamethasone-treated animals compared with trained and metformin-treated rats (Table 1).

**Table:**
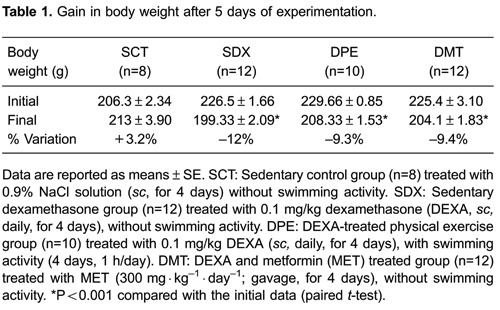

**Figure 2:**
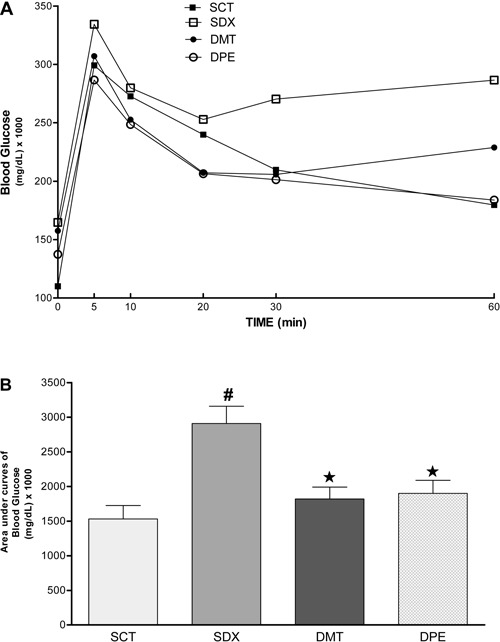
Glucose tolerance test (GTT) carried out in different groups of rats. SCT: Sedentary control group (n=8) treated with 0.9% NaCl solution (*sc*, for 4 days) without swimming activity. SDX: Sedentary dexamethasone group (n=12) treated with 0.1 mg/kg dexamethasone (DEXA, *sc,* daily, for 4 days), without swimming activity. DPE: DEXA-treated physical exercise group (n=10) treated with 0.1 mg/kg DEXA (*sc,* daily, for 4 days), with swimming activity (4 days, 1 h/day). DMT: DEXA and metformin (MET) treated group (n=12) treated with MET (300 mg·kg^−1^·day^−1^; gavage, for 4 days), without swimming activity. *A*, Data are reported as means±SE. *B*, Data are reported as the mean area under the curve±SE for each group. ^#^P<0.001 compared with the SCT group. *P<0.01 compared with the SDX group (ANOVA followed by Tukey's test).

Figure 2A and B shows glucose-concentration profiles during the GTT. Average baseline glucose concentrations (before intravenous injection of glucose) were 110.1±2.25 mg/dL for the SCT group, 164.7±4.45 mg/dL for the SDX group, 157.5±4.16 mg/dL for the DMT group, and 137.4±8 mg/dL for the DPE group. Average glucose values in exercising animals were significantly lower than those in the SDX group. A similar trend was reported 5, 10, 20, 30, and 60 min after intravenous injection of glucose, so physical exercise had a significant effect on decrease in glucose levels.

The DPE group presented a significant decrease in glucose values compared with those of the SDX group during GTT at all time-points, with values similar to those of the control group. These findings suggest that physical exercise through swimming prevented the development of dexamethasone-induced glucose intolerance in Wistar rats. Furthermore, average glucose values in the DPE group were not significantly different compared with those of the DMT group. This finding demonstrated that physical activity can correct dexamethasone-induced hyperglycemia (glucose intolerance) in rats and can improve their glucose profile, as has been observed with metformin.

An increased glucose concentration obtained from the AUC during GTT (Figure 2B) was observed in the SDX group (2909±251.2 mg/dL) compared with that obtained from the SCT group (1531±195.6 mg/dL). In fact, DMT (1821±170.2 mg/dL) and DPE (1901±187.3 mg/dL) groups showed reduced levels of glucose in blood compared with the SDX group.

**Figure 3:**
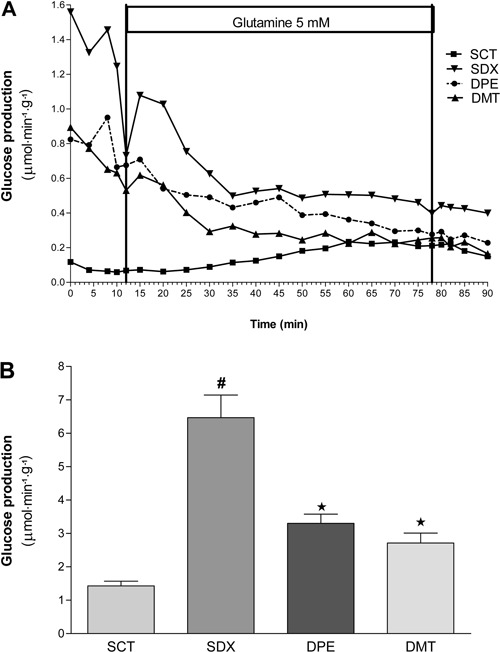
Hepatic production of glucose in different animal groups. SCT: Sedentary control group (n=8) treated with 0.9% NaCl solution (*sc*, for 4 days) without swimming activity. SDX: Sedentary dexamethasone group (n=12) treated with 0.1 mg/kg dexamethasone (DEXA, *sc,*daily, for 4 days), without swimming activity. DPE: DEXA-treated physical exercise group (n=10) treated with 0.1 mg/kg DEXA (*sc,*daily, for 4 days), with swimming activity (4 days, 1 h/day). DMT: DEXA and metformin (MET) treated group (n=12) treated with MET (300 mg·kg^−1^·day^−1^; gavage, for 4 days), without swimming activity. *A*, Data are reported as means±SE. *B*, Data are reported as the mean area under the curve±SE. ^#^P<0.01 compared with the SCT group. *P<0.01 compared with the SDX group (ANOVA followed by Tukey's test).

Given that the livers of fasting rats were used for experiments, L-glutamine served as the gluconeogenic substrate. Liver production of glucose was reduced in the control group at the start of perfusion (first 10 min) because the substrate had not yet been infused (Figure 3A). However, high rates of glucose perfusion were observed in DMT and DPE groups compared with that obtained in the control group (albeit reduced when compared with that of the SDX group). After perfusion of L-glutamine, an increase in glucose concentration was observed and related to gluconeogenesis pathways. This effect was reported in the SDX and DMT groups, but not in control and DPE groups. Five minutes after glutamine infusion, glucose production decreased. However, in the DMT and DPE groups, glucose concentrations were lower than those of the SDX group, and an increase in glucose production was observed in the control group. These data suggest that physical exercise and metformin treatment have protective effects on the onset of acute glucose intolerance and reduce production of glucose in the liver.

Data from the AUC obtained from *in situ* liver perfusion curves (Figure 3B) demonstrated that the SDX group (6.466±0.646) displayed an area that was larger than that obtained in the control group (1.531±195.6). DPE (3.300±0.276) and DMT (2.713±0.296) groups showed similar glucose concentrations to those obtained from the metformin-treated trained group, suggesting that physical exercise reduced glucose production in the liver and glucose intolerance with the same efficacy after metformin treatment.

## Discussion

The present study sought to compare the acute effects of aerobic exercise and metformin treatment on glycemic control in Wistar rats. We aimed to validate the literature and provide comparative experimental evidence using a modern anti-diabetic biguanide that is the first-line choice in DM treatment. To date, various animal models have been used to study DM and its therapies, with some treatments producing negative side effects. For instance, alloxan- and streptozotocin-induced treatments have revealed irreversible lesions in pancreatic beta cells, thereby promoting failed production of insulin and diabetic status (4,20). In many experimental studies (15-20,25,26), acute and chronic adaptations to physical exercise have been demonstrated. However, few studies have been designed to specifically compare the protective effects of physical exercise and metformin on acute hyperglycemia induced by dexamethasone. Furthermore, gain in body weight was determined every day throughout the study period. However, an increase in body weight was observed only in the control group; a significant decrease was noted in the other groups. Collectively, however, the present study corroborates findings from other investigations suggesting that dexamethasone treatment induces a decrease in the body weight of exposed animals (27,28). The reducing effect dexamethasone treatment has on body weight has been stated to occur (at least in part) through several related factors: suppression of synthesis of muscle protein; increased protein catabolism; increased energy expenditure; decreased intake of food (29,30). The mechanisms responsible for glucocorticoid-stimulated metabolic disorders (including those induced by dexamethasone) are not well established. Symptoms associated with such treatment, including insomnia and highly depressive moods, reduced memory, weight loss, and debilitation of the organism, have been reported (31).

The GTT undertaken in the present study suggested that after physical exercise, treated rats and control rats showed a hypoglycemic state similar to those that underwent metformin treatment with respect to glucose tolerance. This finding suggested improved sensitivity to insulin, but we could not confirm quantitatively whether this change resulted from higher levels of insulin production or an improved capacity of insulin-sensitive tissues to uptake substrate. This was a limitation of our investigation. Nonetheless, it has been demonstrated that physical exercise provides immediate metabolic adjustment (acute adaptation) and chronic adjustment after a practice period, thereby suggesting improvements in contraction-mediated insulin sensitivity rather than an augmented insulin response after physical exertion. Exercise requires higher energy demands to maintain homeostasis (3,12,23). Oxygen consumption during exercise increases about 20-fold, and glucose utilization increases 7- to 20-fold, compared with that in non-exercised muscles (22), as may be observed after swimming sessions by rats. In the present study, the effects of physical exercise on acute hyperglycemia may have been related to the reduction in glucose content in tissues, as has been observed in different experimental models of DM (15,16,20). For instance, studies using molecular quantification showed that physical training increases the activity of tyrosine kinase at insulin receptors, glucose transporters in skeletal muscle (glucose transporter type 4), translocation and phosphorylation of substrates at insulin receptors (IRS-1 and IRS-2) and its association with phosphoinositide3-kinase (PI3-kinase) (32). In fact, molecular adaptations for glucose regulation may be related to disease severity (26), as observed in models of dexamethasone-induced acute hyperglycemia presenting with mild metabolic alterations.

Moreover, we report a significant increase in hepatic production of glucose during *in situ* liver perfusion for SDX, trained rats (DPE) and rats treated with metformin (DMT). The increased hepatic levels of glucose observed in the dexamethasone-treated group may have been related to the effect of glucocorticoids. These hormones induce counter-regulatory effects on insulin and exert predominant actions on intermediate metabolism, thereby affecting hepatic, muscle and adipose tissues. The aforementioned class of hormones increases glucose levels, thereby limiting peripheral consumption and production of glucose (33). In addition, glucocorticoids stimulate hepatic gluconeogenesis, metabolize fatty acids and glycerol released from adipocytes, catabolize amino acids from the inhibition of peripheral protein synthesis, and activate phosphoenolpyruvate carboxykinase (rate-limiting enzyme responsible for gluconeogenic events) (34). Hence, with respect to our findings, we suggest that dexamethasone treatment revealed persistent hyperglycemia as a consequence of insulin resistance. Increased glucose levels during liver perfusion of dexamethasone-treated animals were reduced after metformin treatment and physical exercise. High glucose concentrations could be because glucocorticoids have hyperglycemic and lipolytic effects and, therefore, diabetogenic and anti-insulin actions (35,36). Furthermore, glucocorticoids may trigger persistent hyperglycemia induced by glucagon, epinephrine and/or growth hormone (31).

With regard to all the parameters evaluated in the present study (glucose intolerance and hepatic production of glucose), the DPE group presented a glycemic profile similar to that of the DMT group. This finding suggested a preventive effect on hyperglycemia induced by glucocorticoids that is similar to that between metformin and physical aerobic exercise. In general, it is accepted that physical exercise triggers metabolic alterations and facilitates glucose transport into cells. Regular physical exercise is important for glucose control in insulin-resistant individuals. Silveira et al. (37) reported that low exercise intensity for long periods affects glucose control in non-insulin-dependent diabetic individuals. Moreover, insulin economy evaluated during the GTT in overweight and obese individuals who are active and subject to different intensities and duration of exercise has shown improved results compared with those obtained in a sedentary group (38). Collectively, physical exercise is an optimal method to enhance insulin economy and improve glycemic control in healthy and compromised individuals.

Trained animals reduced their glucose levels after a GTT and liver perfusion compared with those in the dexamethasone group. In addition, high blood and perfusate concentrations of glucose in dexamethasone-treated sedentary rats were observed, suggesting that regular physical exercise increased glucose uptake in peripheral and hepatic tissues. These findings demonstrate a beneficial effect of physical training for the improvement of insulin sensitivity. However, it is not clear which molecular mechanisms are involved in the increased uptake of glucose by muscles. Further studies must be completed to explain how the intensity, duration, and magnitude of exercise differentially affect insulin-signaling pathways in experimental models of acute hyperglycemia (12). We speculate, however, that hemodynamic changes induced by physical exercise may be involved because a single exercise session reduced sympathetic activity and increased muscle blood flow in the post-exercise period compared with non-exercising controls. In fact, after a single exercise session, hyperinsulinemia promotes diminished sympathetic activity even though increased vasodilatation in muscles is observed as a consequence of hemodynamic changes and insulin resistance is improved (39).

Increased sensitivity to insulin observed after physical training seems to be a consequence of an improved response of the insulin receptor to events occurring downstream, specifically phosphorylation of proteins related to insulin signaling hormones. The transduction signal at insulin receptors IRS-1 and IRS-2 coupled with PI3-kinase activity is increased in the skeletal muscles of trained rats and in non-diabetic, insulin-resistant and DM2 humans (40). The exercise protocol used in the present study is, therefore, a contribution to the literature because it avoids glucose intolerance by improving insulin sensitivity in trained dexamethasone-treated animals. This phenomenon may be because exercise practice promotes increased expression of glucose transporters and up-regulates the enzyme activity linked to their metabolism. These two phenomena are related to improved glucose tolerance (40). Conversely, moderate exercise affects glucose metabolism in muscles and the glycogen-synthase activity that may occur without alterations in insulin-signaling cascades (40). Additionally, physical exercise promotes the transport of glucose through increased levels of insulin-like growth factor-1 without increasing the number of transporters in rats submitted to training for 8 weeks (26).

Collectively, there are numerous, complex, and dynamic metabolic/molecular pathways involved with improved tolerance to glucose because the latter is related to exercise and glucose transport (32). Many studies have shown the additional effects of insulin action and muscle contraction, and suggest that insulin and exercise activate glucose transport by different mechanisms (32).

Physical exercise (swimming) may be a viable form of therapy to prevent development of glucose intolerance induced by dexamethasone, and may be comparable with the effects of metformin. Physical exercise and metformin restored glucose tolerance, thus our results suggest that exercise practice is a preventive activity that avoids the development of glucose intolerance and DM2 onset if glucocorticoid therapies are used. Our results suggest that the anti-diabetic medication metformin has similar efficacy to physical exertion, without the added benefit of improved cardiorespiratory fitness.
